# Two-Dimensional Fluorescence Difference Spectroscopy to Characterize Nanoparticles and their Interactions

**DOI:** 10.1038/srep33287

**Published:** 2016-09-14

**Authors:** Miranda N. Hurst, Robert K. DeLong

**Affiliations:** 1 Nanotechnology Innovation Center Kansas State, Department of Anatomy and Physiology, College of Veterinary Medicine, Kansas State University, Manhattan, KS 66506, USA

## Abstract

Two dimensional fluorescence difference spectroscopy (2D FDS) detects nanoparticle interactions following surface functionalization and biomolecule loading by generating a spectral signature of the fluorescent intensity per excitation and emission wavelengths. Comparing metal oxide nanoparticles revealed a unique spectral signature per material composition. 2D FDS showed to be sensitive to changes in surface properties between ZnO NPs synthesized by different methods. ZnO NP loaded with glycol chitosan, polyacrylic acid (PAA), or methoxy polyethylene glycol (mPEG) exhibited a distinct spectral signature shift. ZnO NP loaded with Torula Yeast RNA (TYRNA)(640 nm), polyinosinic: polycytidylic acid (pIC)(680 nm), or splice switching oligonucleotide (SSO)(650 nm) each revealed a shift in emission. Ras-Binding domain (RBD) at three concentrations (25, 37.5, 50 μg/mL) showed that fluorescent intensity was inversely related to the concentration of protein loaded. These data support 2D FDS as a novel technique in identifying nanoparticles and their surface interactions as a quality assurance tool.

Nanotechnology is a rapidly developing field that has caught the interest of the scientific community due to its potential applications in biomedical research. Nanomaterials are being synthesized from nearly every element creating unique particles that vary in composition, size, shape, and crystal arrangement^1^,^2^,^3^,^4^,^5^,^6^,^7^. This leads to a wide array of nanoparticles with physico-chemical properties including magnetism, electronics, and optics. The large surface area to volume ratio that nanoparticles offer makes them suitable for surface functionalization allowing for attachment of targeting or therapeutic molecules^8^. Nanomaterials are typically less than 100 nm in diameter, making them small enough to penetrate mammalian cells. When nanoparticles are delivered systemically, attached targeting molecules enable detection of certain cell populations, while attached therapeutic compounds can treat targeted cells. However, while nanomaterials are such a promising platform for biomedical and industrial applications, additional characterization methods are needed to confirm complexation during drug formulation.

Currently there are a limited number of techniques available to characterize nanoparticles and their interactions. Nanoparticles are visualized with transmission and scanning electron microscopies^9^,^10^, which can be used to quantify particle size and uniformity along with dynamic light scattering (DLS)^11^. X-ray photoelectron spectroscopy (XPS) has been shown to elucidate the molecular composition at the particle’s surface^12^. Surface functionalization of a nanoparticle is validated by FT-IR and NMR^13^, where nanobio interactions are further characterized by UV- visible absorbance, fluorescence, and circular dichroism (CD) spectroscopies^11^,^14^.

Applications of fluorescence spectroscopy are currently restricted to time- resolved fluorescence^15^, Förster resonance energy transfer (FRET)^16^, and fluorescence polarization^17^. However, these techniques often require the use of a dye to probe the samples, complicating the measurement as release or decay of the dye must be considered. 2D FDS is an application of fluorescence spectroscopy that detects the intrinsic fluorescence of the nanomaterial, and thus does not rely on the attachment of a fluorophore. Similarly, FT-IR, NMR, and XPS are elaborate techniques that not only require expensive instrumentation, but require additional software analysis of the peaks. Whereas, 2D FDS is easy to perform on a standard plate reader equipped with fluorescence detectors and does not require complex data analysis. There is a need for validating the presence of the biomolecule binding the nanoparticle surface before performing an in depth study on the mechanism of interaction or prior to delivery into tissue culture and animal models. Therefore, we propose 2D FDS as a quality assurance technique capable of confirming complexation between a nanomaterial and molecule.

When light of sufficient energy (E > Eg) is incident on a material, photons are absorbed and electrons become excited, moving the electrons from the valence band to the conduction band leaving behind holes. Some of the absorbed energy is lost to vibration, however, radiative relaxation results in the emission of fluorescent light as the electron returns to its ground state^18^. Fluorescence emission is thus a result of the band gap in semi-conductor materials, or the difference in energy between the valence band and the conduction band. This is specifically important as the continuous energy band gap breaks down further into discrete energy levels at the nanoscale. These discrete energy levels can provide unique properties that are not observable macroscopically^19^. Further, the band gap is related to the particle size, where an increase in the band gap occurs as the particles approach quantum confinement^20^.

Upon electrostatic interaction, the optical properties within the nanomaterial change, resulting in a shift in the peak fluorescence excitation and emission wavelengths, or spectral signature. Two dimensional fluorescence difference spectroscopy (2D FDS) yields a spectral signature by plotting the excitation against emission wavelengths and identifying intersects of high intensity. Here we demonstrate 2D FDS as a method to identify nanomaterials and confirm complexation with molecules following surface functionalization or biomolecule loading.

## Materials and Methods

Commercial zinc oxide nanoparticles (ZnO NP) and HPLC grade water were purchased from Sigma Aldrich (St. Louis, MO, USA). Iron oxide nanoparticles (Fe_2_O_3_ NP) were obtained from Plasma Chem (Berlin, Germany). Nickel oxide nanoparticles (NiO NP) were synthesized by Dr. K. Ghosh (Missouri State University, Springfield, MO, USA). ZnO NP synthesized by microwave irradiation methods by G. Glaspell has been communicated elsewhere. ZnO nanobelts were synthesized as previously reported^21^. Glycol chitosan was obtained from MP Biochemicals. Poly acrylic acid (PAA), methoxy polyethylene glycol 5000 (mPEG 5000), torula yeast RNA (TYRNA), and polyinosinic: polycytidylic acid (pIC) were purchased from Sigma Aldrich. Splice switching oligonucleotide (SSO) was synthesized by Trilink Biotechnologies (San Diego, CA, USA). The 623 SSO sequence (5′-GTT ATT CTT TAG AAT GGT GC- 3′) was modified with a phosphothioate backbone and 2′-O Methyl groups^22^. Ras-Binding domain (RBD) was purchased from EMD Millipore. Samples were read in a black 96 well microplate (Midsci).

Nanoparticles are weighed out at 1–2 mg and suspended in 1 mL of HPLC grade water in sterile microcentrifuge tubes. The particles were dispersed for 5 minutes at 50% amplitude and 20 seconds pulses using Sonics Vibracell VCX130 probe ultra- sonicator. For nanoparticle only samples, a concentration of 1 mg/mL or 0.5 mg/mL was scanned at a volume of 200 μL in a black 96 well plate. For surface functionalization studies, a concentration of 1 mg/mL nanoparticle was incubated with 0.1 mg/mL polymer, either glycol chitosan, PAA, or mPEG, for 30 minutes at room temperature on an orbital shaker set at 250 rpm prior to being transferred to a black 96 well plate. For RNA loading experiments, 1 mg/mL nanoparticle was incubated with 0.1 mg/mL RNA, either TYRNA, pIC, or SSO, on the orbital shaker set to 250 rpm for 30 minutes while on ice then transferred to a black 96 well plate. For RBD studies, the nanoparticle was diluted to a concentration of 0.5 mg/mL, and RBD concentrations included 50 μg/mL, 37.5 μg/mL, and 25 μg/mL. The nanoparticle and protein were incubated for 30 minutes on the orbital shaker set to 250 rpm at room temperature then transferred to a black 96 well plate. Microplates were scanned using the Molecular Devices Spectramax i3x spectrophotometer utilizing the Spectral Optimization Wizard as part of Softmax Pro 6.4.2 software (Sunnyvale, CA, USA). Settings were specified to read fluorescence end point of unknown wavelengths, prompting the Spectral Optimization Wizard. The range of excitation and emission wavelengths to scan was inputted as 250–500 nm and 280–700 nm, respectively. Read height (from the top of the microplate) was set to 1 mm, and 5 or 10 nm wavelength increments were selected. The flash lamp was set to 6 pulses, where the values for each excitation and emission intersect were averaged between 6 readings. The software inherently analyzes the data using the equation: (S-B)/B where the difference between the sample and background readings were divided by the background.

## Results

**Figure 1:**
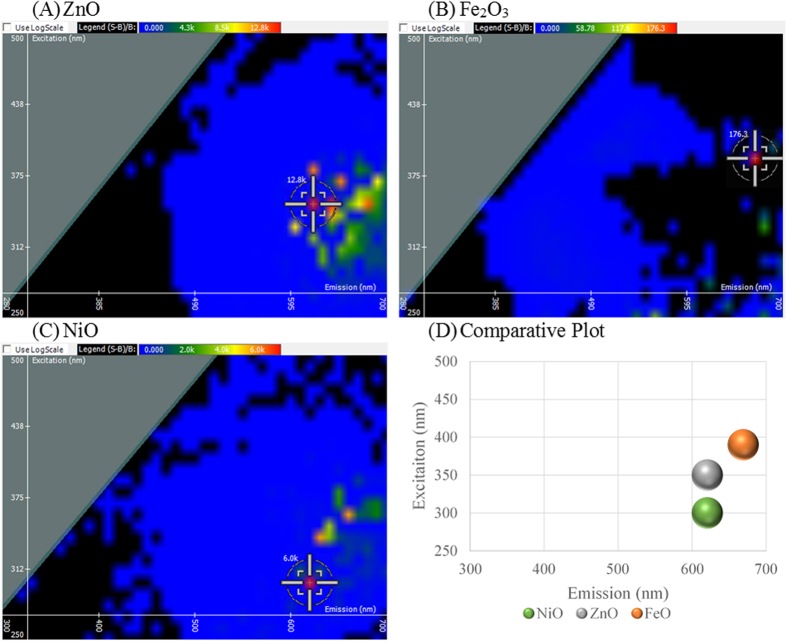
Spectral Signature is dependent upon Material Composition. 2D FDS of (**A**) zinc oxide (ZnO), (**B**) iron oxide (Fe_2_O_3_), and (**C**) nickel oxide (NiO).

Zinc oxide (ZnO) is an indigenously fluorescent material that is of interest due to its wide range of conductivity and its applications for electronic and photonic modalities. ZnO has a wide band gap energy of 3.37 eV^23^, which calculates to 375 nm as the start of emission. Due to the Stoke’s shift phenomenon, the optimal excitation wavelength of ZnO is between 325–350 nm. In Fig. 1(A), ZnO exhibited an optimal excitation and emission intersect of 350 nm and 620 nm, respectively, with a fluorescent intensity of 12,800 light units. Iron oxide (Fe_2_O_3_) is predominantly exploited for its magnetic properties and its high thermodynamic stability. Direct electron transitions for hematite (α-Fe_2_O_3_) can be observed at the band gap energies of 3.0–4.7 eV^24^, which corresponds to a range of excitable wavelengths from 215–390 nm. In Fig. 1(B), Fe_2_O_3_ gives an optimal excitation wavelength of 390 nm with emission at 670 nm and a fluorescent intensity of 176.3 light units. Nickel oxide (NiO) nanocrystals were shown to have a band gap energy of 3.62 eV^25^. This exciton ground state energy corresponds to a range of wavelengths from 290–315 nm. In Fig. 1(C), NiO gives a spectral signature showing optimal excitation at 300 nm with an emission at 620 nm and a fluorescent intensity of 6,000 light units. Evaluating the optimal excitation and emission intersects for the three nanomaterials revealed that this technique is specific to a material’s elemental composition and dependent upon the band gap energy within the material. The unique spectral signatures can be seen on the comparative plot of the three nanoparticles in Fig. 1(D).

**Figure 2:**
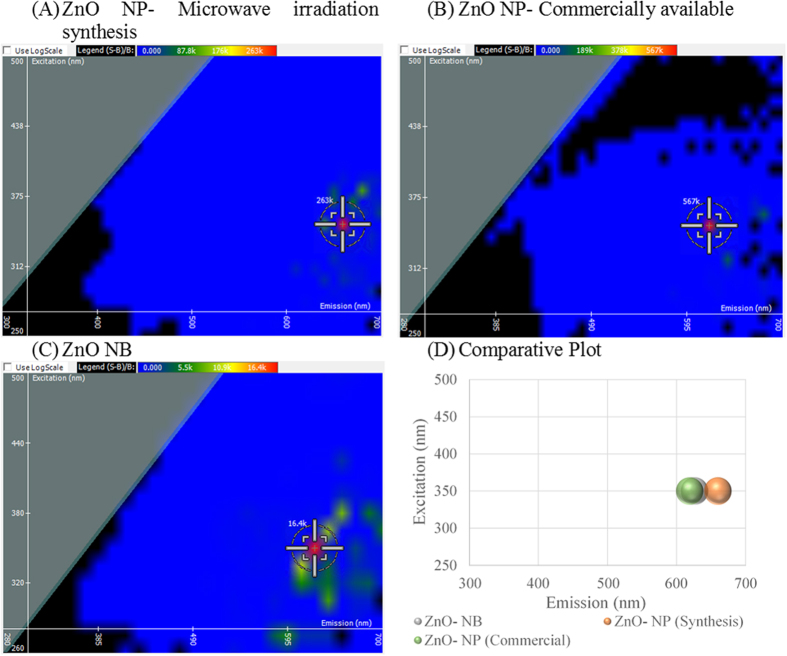
Comparing Synthesis Methods and Nanomaterial Morphology on the Spectral Signature of zinc oxide (ZnO). (**A**) ZnO nanoparticle (NP) synthesized by microwave irradiation, (**B**) ZnO NP purchased commercially, (**C**) ZnO nanobelts (NB), and (**D**) comparative plot of the three ZnO species.

To further explore how this technique can be applied to characterize nanomaterials, we compared two types of ZnO nanoparticles differing in surface properties, where one was derived commercially and the other synthesized by microwave irradiation (M.I.) method. These nanoparticles were compared to ZnO nanobelts, or long filamentous nanostructures, to observe the effects of morphology on the spectral signature. In Fig. 2(A), the synthesized ZnO NP gave an optimal excitation and emission intersect at 350 and 660 nm with an intensity of 263,000 light units. In Fig. 2(B), the commercial ZnO NP yields an identical excitation of 350 nm and emission at 620 nm. The fluorescent intensity of the commercial ZnO NP is twice that of the synthesized ZnO NP at 567,000 light units. In Fig. 2(C), ZnO nanobelts give an optimal excitation and emission intersect of 350 nm and 625 nm with a fluorescent intensity of 16,400 light units. When graphed comparatively in Fig. 2(D), it becomes evident that the excitation wavelength reflects the materials composition. However, the emission wavelength differs between the two ZnO nanoparticles resulting from differences in surface electronic properties. The fluorescent intensity of the three ZnO nanomaterials differ significantly, revealing that synthesis as well as morphology play largely into optimizing the fluorescent signal of the nanoparticle for detection.

**Figure 3:**
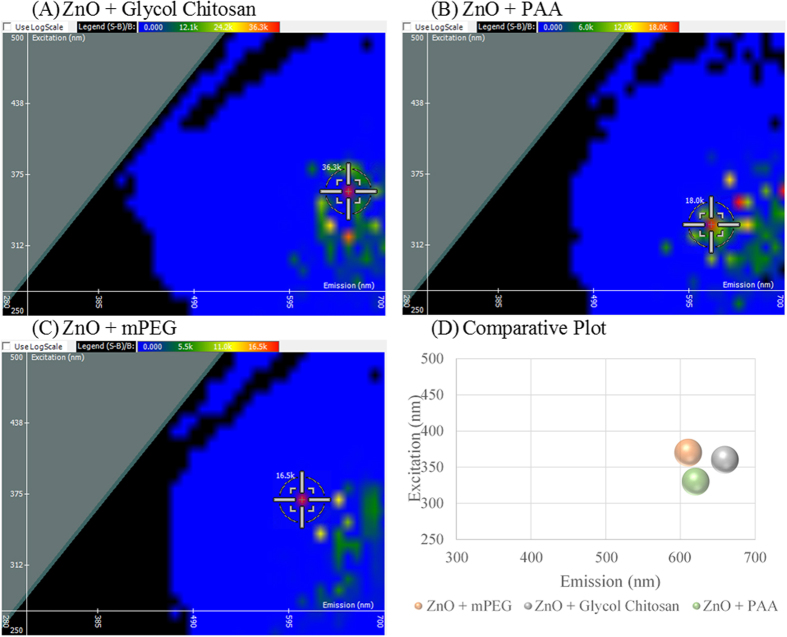
Spectral Signature Shift upon Surface Functionalization of zinc oxide (ZnO) nanoparticle as a result of polymer association. (**A**) ZnO bound to glycol chitosan, (**B**) ZnO coated with poly(acrylic acid) (PAA), (**C**) ZnO interacting with methoxy poly(ethylene glycol) (mPEG), and (**D**) a comparative plot of the three polymer coatings.

Nanoparticles offer a high surface area to volume ratio making them ideal delivery agents and prone to surface functionalization^26^,^27^. With such vast applications of creating complex nanocarriers housing multiple molecules, 2D FDS lends itself to confirm complexation through detection of changes in the fluorescent signature. As shown in Fig. 2(B), ZnO NP gives an optimal excitation and emission intersect of 350 nm and 620 nm with a fluorescent intensity of 567,000 light units. This optimal intersect shifts upon ZnO NP binding polymers: glycol chitosan, polyacrylic acid (PAA), or methoxy polyethylene glycol (mPEG). Glycol chitosan is of interest due to its biocompatibility and biodegradability, as well as the ability to covalently link biomolecules to the amine groups that derive its backbone^28^. In the presence of glycol chitosan shown in Fig. 3(A), the spectral signature shifts to 360 nm and 660 nm, respectively. Fluorescent quenching was observed upon interaction with glycol chitosan, diminishing the intensity by over 530,000 light units. PAA has been used to prevent nanoparticle aggregation and serves as a platform to electrostatically load biomolecules^29^. In the presence of PAA shown in Fig. 3(B), an excitation shift to 330 nm is observed, with the emission wavelength remaining unchanged at 620 nm. Again there is significant fluorescent quenching indicative of interaction with a difference of 549,000 light units. As a copolymer nanoparticle, mPEG has been used to encapsulate therapeutics, offering a slow release, while maintaining particle stability *in vivo*^30^,^31^. With an even greater decrease in intensity, mPEG shows a shift in excitation to 370 nm and a minor shift in emission to 610 nm in Fig. 3(C). A comparative plot is given in Fig. 3(D) revealing three distinct shifts in the spectral signature of ZnO NP per polymer.

**Figure 4:**
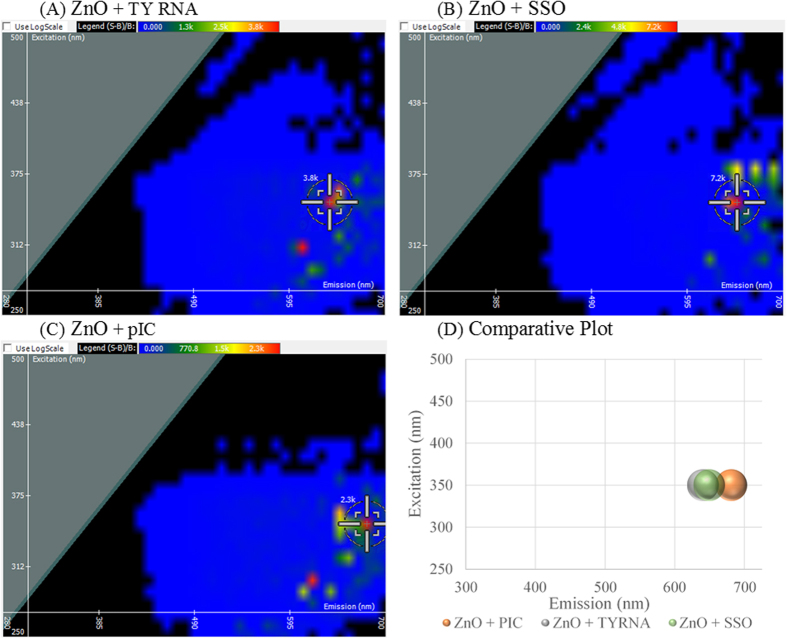
Spectral Signature Shift upon RNA loading onto zinc oxide (ZnO) nanoparticle. ZnO loaded with (**A**) Torula Yeast RNA (TYRNA), (**B**) splice switching oligonucleotide (SSO), and (**C**) polyinosinic: polycytidylic acid (pIC). A comparative plot is shown in (**D**).

Surface functionalization of nanoparticles is not limited to chemicals and synthetic compounds, but rather a large aim of biomedical nanotechnology focuses on the loading of biomolecules including protein, DNA, and RNA onto nanoparticles^32^,^33^,^34^. Typically RNA cannot be detected by fluorescence spectroscopy without the aid of a dye, however, 2D FDS can further be used to detect successful RNA adsorption to the nanoparticle surface. Compared to the spectral signature of ZnO NP shown in Fig. 1(A), a shift is observed in the optimal emission wavelength of the three RNAs shown in Fig. 4. RNA derived from Torula yeast (TYRNA) was used as a model of RNA found naturally in the biological environment. Upon binding ZnO NP, a shift in the emission of the spectral signature is observed at 640 nm, while the excitation wavelength remains unchanged as seen in Fig. 4(A). We have previously reported dendrimer and nanoparticle- mediated delivery of splice- switching oligonucleotides (SSOs) as a means to test the RNA functionality upon transport into the cell^35^. A shift in the optimal emission wavelength, to 650 nm, is observed when ZnO NP binds SSO, while excitation remains unaltered as shown in Fig. 4(B). Polyinosinic-polycytidylic acid (pIC) is a double stranded RNA that initiates an innate immune response by binding the intracellular receptor, toll- like receptor 3 (TLR3)^36^. PIC inhibits tumor growth by recruiting mature dendritic cells to the tumor site, overcoming immunosurveillance evasion^37^. There is a large interest in the scientific community in loading vaccine adjuvants onto nanoparticles; therefore, pIC was chosen representatively. The greatest shift in emission was observed for pIC to 680 nm as shown in Fig. 4(C). A comparative plot is given in Fig. 4(D), where ZnO NP bound to TYRNA and SSO nearly overlap in the shifted spectral signature, and pIC bound to ZnO NP is shown to have the greatest shift. Dynamic fluorescent quenching is observed suggesting complexation as the intensity decreased from 12,800 light units of ZnO NP alone to 2,300–7,200 light units in the presence of RNA. This is consistent with the direct RNA interaction to ZnO NP as evidenced by Raman spectroscopy^38^.

**Figure 5:**
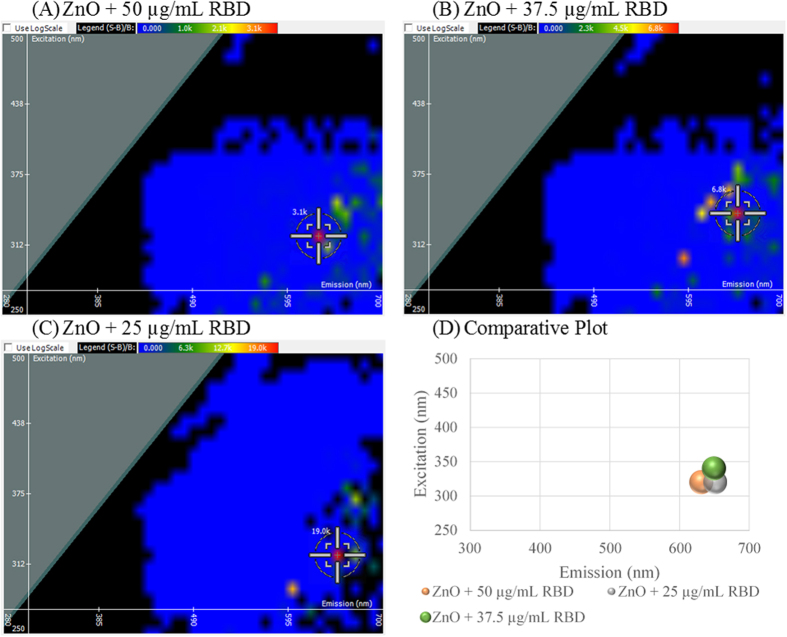
Spectral signature shifts upon ZnO NP interaction with Ras-Binding domain (RBD) peptide at varying concentrations 50 μg/mL (**A**), 37.5 μg/mL (**B**), and 25 μg/mL (**C**). A comparative plot is shown in (**D**).

2D FDS can be used to detect protein interactions at the nanoparticle surface. Ras-Binding domain (RBD) contains the first 149 residues of C-Raf-1 protein which binds Ras-GTP, a known cancer target, as a downstream effector^39^,^40^. RBD was loaded onto ZnO NP at three concentrations as shown in Fig. 5. Compared to ZnO NP in Fig. 1(A), a shift in excitation and emission, from 350 nm and 620 nm respectively, was observed for the three spectral scans. At 50 μg/mL RBD, shown in Fig. 5(A), the excitation shifted to 320 nm and the emission shifted to 630 nm. This concentration revealed the greatest fluorescent quenching from 12,800 relative light units to 3,100 light units. At 37.5 μg/mL RBD, the spectral signature of ZnO NP shifted to a different excitation and emission intersect, 340 nm and 650 nm, respectively. As shown in Fig. 5(B), fluorescent quenching was observed, to a lesser extent, with relative light units of 6,800. At 25 μg/mL RBD, shown in Fig. 5(C), the excitation shifted to 320 nm, and the emission shifted to 350 nm. At this concentration, fluorescent enhancement was observed increasing the relative light units to 19,000. A comparative plot is given in Fig. 5(D) revealing that excitation shifts towards the ultra-violet end of the spectrum and emission increases towards the infrared end of the spectrum upon interaction of the protein.

All values obtained from the spectral signatures shown throughout this manuscript are listed in Table 1. ZnO NP gives the highest fluorescent intensities (12,800–567,000 RLU) compared to other nanomaterials. Interaction of a polymer, RNA, or protein at the nanoparticle surface causes a decrease in the intensity and a shift in the excitation (320–370 nm) and emission (610–680 nm).

In conclusion, 2D FDS generated a fluorescent signature dependent upon material composition. Three metal oxide nanoparticles, including zinc, iron, and nickel, are shown representatively; however, this technique could be used to determine the optimal fluorescent excitation and emission wavelengths for other nanomaterials and those derived from other polymeric or biomaterials (data not shown).

2D FDS can further discriminate between the surface properties of nanoparticles. It is known that heating and cooling cycles, along with differing the gaseous or solvent environment during synthesis, result in nanomaterials with varying surface properties. When comparing ZnO synthesized by microwave irradiation to commercial ZnO, a shift in emission was evident, which supports that the electronic properties at the nanomaterial surface change as a result of the synthesis process. However, no shift was evident when comparing ZnO nanobelts to commercial ZnO nanoparticles. Further investigation is required to conclude the effects of morphology on the spectral signature of ZnO nanomaterials. However, this data supports the conclusion that the optimal fluorescent excitation and emission intersect generated by 2D FDS is a result of the material composition. Comparing commercial ZnO NP at two concentrations, 0.5 and 1 mg/mL, shows a significant decrease in fluorescence from 567,000 light units to 12,800 light units. Therefore, it can be deduced that the fluorescent intensity of the nanomaterial is concentration-dependent.

When considering surface functionalization of a nanoparticle, a shift in the excitation wavelength can often be negated as materials are excitable within a range of wavelengths. However, the emission wavelength is often fixed per excitation wavelength, meaning that changes in the emission wavelength are indicative of surface interactions between the nanoparticle and molecule. This could be due to energy transfer between the adjacent electron orbitals of the interacting molecules and the formation of intermediate band gap levels. However, when assessing the extent of fluorescent quenching, mPEG exhibited the greatest quenching followed by PAA and glycol chitosan. Because PAA exhibits no shift in emission and intermediate fluorescent quenching, it can be concluded that it minimally binds to ZnO NP. Similarly, mPEG reveals a minor emission shift and high fluorescent quenching, indicating interaction to ZnO NP. Glycol chitosan gives the greatest emission shift, but the least fluorescent quenching, indicating binding to ZnO NP by a different mechanism. Each polymer loaded onto ZnO NP gave a distinctive shift in the spectra which could be attributed to the type of interaction between the polymer and nanoparticle. However, further investigation is required to determine the interaction mechanism between each polymer and ZnO NP.

Assessing biomolecule loading onto the nanoparticle surface, it was shown that ZnO NP binds RNA specifically as TYRNA, pIC, and SSO each gave a unique shift. PIC bound ZnO NP with the highest affinity as it gave the greatest shift in emission and fluorescent quenching. SSO gave an intermediate emission shift, followed by TYRNA which shifted the least by 20 nm. The trend observed for fluorescent quenching (pIC > TYRNA > SSO) could be attributed to the RNA size and surface area coverage at the ZnO NP surface. SSO is a short 20 nucleotide single stranded RNA with a linear structure. TYRNA is a single stranded, long RNA molecule that undergoes complex tertiary folding similar to tRNA. PIC is double stranded polymer RNA with a helical structure similar to DNA. Therefore, pIC being the largest molecule quenched the greatest, and SSO being the smallest molecule quenched the least. The length and varying structures of the three RNAs chosen attributes to the differences in affinity for ZnO NP resulting in a unique shift.

ZnO NP loaded with three concentrations of RBD revealed that the excitation undergoes a blue shift and the emission undergoes a red shift upon interaction. This indicates that energy is lost to other forms such as vibration than to fluorescent emission of photons as the emission shifts to longer wavelengths. It can be further concluded that the fluorescent intensity given from 2D FDS is inversely related to the concentration of protein interacting at the nanoparticles surface. The greater the protein quantity, the greater the quenching as more protein is present at the nanomaterial’s surface. At small protein concentrations, fluorescent enhancement occurs as a result in the increase in oxygen vacancies at the nanoparticle’s surface upon protein interaction, suggesting interaction with zinc.

Once the range of excitation and emission wavelengths are established for the nanomaterial of interest, surface functionalization and biomolecule loading onto the nanoparticle can further be detected by 2D FDS. In the presence of the polymers or biomolecules, a distinct shift was observed in the spectral signature of ZnO NP per species and concentration loaded onto the particle. The greater the quenching of fluorescent intensity and the greater the shift in emission correlate with stronger binding. 2D FDS is a novel technique that provides a larger understanding on the optical properties of nanoparticles, while validating interactions at the nanomaterials surface. 2D FDS lends itself as a quality assurance tool that could be extended to other intrinsically fluorescent molecules.

## Additional Information

**How to cite this article**: Hurst, M. N. and DeLong, R. K. Two-Dimensional Fluorescence Difference Spectroscopy to Characterize Nanoparticles and their Interactions. *Sci. Rep*. **6**, 33287; doi: 10.1038/srep33287 (2016).

## Figures and Tables

**Table 1:** Summary of 2D FDS Values.

Sample	Excitation (nm)	Emission (nm)	Intensity (RFU)
ZnO NP (Commercial, 0.5 mg/mL)	350	620	12,800
Fe_2_O_3_ NP	390	670	176.3
NiO NP	300	620	6,000
ZnO NP (M.I. Synthesis)	350	660	263,000
ZnO NP (Commercial, 1 mg/mL)	350	620	567,000
ZnO NB	350	625	16,400
ZnO + Glycol Chitosan	360	660	36,300
ZnO + PAA	330	620	18,000
ZnO + mPEG	370	610	16,500
ZnO + TYRNA	350	640	3,800
ZnO + pIC	350	680	2,300
ZnO + SSO	350	650	7,200
ZnO + 50 μg/mL RBD	320	630	3,100
ZnO + 37.5 μg/mL RBD	340	650	6,800
ZnO + 25 μg/mL RBD	320	650	19,000

Zinc oxide nanoparticle (ZnO NP), iron oxide nanoparticle (Fe_2_O_3_ NP), nickel oxide nanoparticle (NiO NP), microwave irradiation (M.I.), zinc oxide nanobelt (ZnO NB), polyacrylic acid (PAA), methoxy polyethylene glycol (mPEG), torula yeast RNA (TYRNA), polyinosinic: polycytidylic acid (pIC), splice switching oligonucleotide (SSO), Ras-Binding domain (RBD).
